# Validation of Polish-Language Questionnaires for Assessing the Quality of Life of Patients with Primary Ciliary Dyskinesia (PCD-QOL)

**DOI:** 10.3390/arm92040025

**Published:** 2024-06-24

**Authors:** Magdalena Anita Roszak, Anna Bręborowicz, Aleksandra Szczepankiewicz, Marcin Mikoś, Zuzanna Bukowy-Bieryłło, Barbara Więckowska, Laura Behan, Hanna Dmeńska, Joanna Goździk-Spychalska, Agata Nowicka, Ewa Sapiejka, Paulina Famulska, Elżbieta Gąsecka, Andrzej Pogorzelski, Irena Wojsyk-Banaszak

**Affiliations:** 1 Department of Pulmonology, Pediatric Allergy and Clinical Immunology, Poznan University of Medical Sciences, ul. Szpitalna 27/33, 60-567 Poznan, Poland; abreborowicz@wp.pl (A.B.); marcin@mikos.ws (M.M.); iwojsyk@ump.edu.pl (I.W.-B.); 2 Laboratory of Molecular and Cell Biology, Poznan University of Medical Sciences, ul. Szpitalna 27/33, 60-576 Poznan, Poland; alszczep@ump.edu.pl; 3 Department of Molecular and Clinical Genetics, Institute of Human Genetics, Polish Academy of Sciences, ul. Strzeszynska 32, 60-479 Poznan, Poland; zuzanna.bukowy-bieryllo@igcz.poznan.pl; 4 Department of Computer Sciences and Statistics, Poznan University of Medical Sciences, ul. Rokietnicka 7, 60-806 Poznan, Poland; basia@ump.edu.pl; 5 Primary Ciliary Dyskinesia Centre, NIHR Biomedical Research Centre, University Hospital Southampton NHS Foundation Trust, Tremona Rd, Southampton SO16 6YD, UK; l.behan@soton.ac.uk; 6 Clinic of Pulmonary Diseases, The Children’s Memorial Health Institute, Aleja Dzieci Polskich 20, 04-730 Warsaw, Poland; h.dmenska@gmail.com; 7 Department of Pulmonology, Allergology and Respiratory Oncology, Poznan University of Medical Sciences, ul. Szamarzewskiego 84, 60-569 Poznan, Poland; jogozspych@gmail.com (J.G.-S.);; 8 Department of Pediatrics and Cystic Fibrosis, Polanki Children’s Hospital, ul. Polanki 119, 80-308 Gdansk, Polandp.famulska@gmail.com (P.F.); 9 Department of Pediatrics, Allergology and Immunology, Polanki Children’s Hospital, ul. Polanki 119, 80-308 Gdansk, Poland; e.gasecka@szpitalpolanki.pl; 10 Department of Pneumology and Cystic Fibrosis, Institute of Tuberculosis and Lung Diseases, ul. Rudnika 3B, 34-700 Rabka-Zdrój, Poland; apogorzelski@igrabka.edu.pl

**Keywords:** primary ciliary dyskinesia, quality of life questionnaires

## Abstract

**Highlights:**

**What are the main findings?**

- The quality of life of primary ciliary dyskinesia patients is assessed differently depending on the patient’s country of origin.
- QOL-PCD questionnaires can be used during routine follow-up visits.

**What are the implications of the main findings?**

- The Polish version of the quality of life questionnaire is a simple and feasible tool to assess PCD patients of all ages.
- Polish PCD patients rate their quality of life lower than English-speaking patients.

**Abstract:**

In recent years, questionnaires were published in English to assess the quality of life of patients with PCD (Primary Ciliary Diskinesia) for adults, adolescents aged 13–17 years, and children aged 6–12 years and their caregivers. This study aimed to prepare Polish versions of the questionnaires and validate them in specific age groups with the participation of Polish patients with PCD. The individual questionnaires were translated and discussed with the involvement of the creator of the original questionnaire in English. Patients completed the questionnaires according to their affiliation with one of the groups. Validation was based on internal consistency analysis (Cronbach’s alpha coefficient and split-half reliability) and test–retest reliability (intraclass correlation coefficient—ICC). The internal consistency of all questionnaires was from moderate to very good (Cronbach’s alpha 0.67–0.91, split-half reliability 0.53–0.95). The consistency of the measurements showed excellent repeatability (ICC 0.67–0.91). The surveyed Polish PCD patients rated their quality of life quite well (63–77%). QOL questionnaires for patients with PCD can be used routinely during each medical check-up as a simple tool to provide the doctor with an indication of the effectiveness of treatment and the impact of the disease on the patient’s quality of life.

## 1. Introduction

Primary ciliary dyskinesia (PCD) is a rare genetic disease with an incidence of 1 in 10–20,000 births. The disease is caused by gene mutations encoding proteins responsible for the normal structure and function of motor cilia (ciliopathy) [1]. Motor cilia are present in many organs and systems, including the respiratory tract, the auditory system, the reproductive system, and the embryonic node during embryogenesis. Most often (in 80% of PCD cases), the first symptoms in the form of respiratory distress syndrome (RDS) are observed in the neonatal period, with typical atelectatic lesions of the middle and upper lung lobes revealed in chest X-ray [2]. Abnormal functioning of the respiratory ciliated cells leads to mucus retention in the airways, which is the cause of recurrent infections and chronic cough, and over time, leads to permanent airway damage in the form of bronchiectasis. Patients present the so-called nasal speech caused by nasal blockage, chronic sinusitis, and nasal cavity polyps. Dysfunction of the ear cells of the auditory system results in recurrent otitis media. It can lead to severe complications, including perforation of the eardrum, erosion of the ossicles, and the formation of cholesteatoma. Hearing loss affecting the youngest patients can lead to impaired child speech development. Disorders of the function of the cilia in reproductive system cells reduce fertility in most women and all affected men. Motor cilia are essential for the correct heart and viscera positioning in fetal life. Their dysfunction can cause the abnormal localization of organs, which occurs in about 50% of the patients with PCD. The triad of symptoms visceral inversion, chronic sinusitis, and bronchiectasis is called Kartagener’s syndrome.

The multitude of symptoms that are the clinical manifestation of PCD and the therapeutic management that includes systematic daily pulmonary rehabilitation and inhaled and oral medications can reduce the quality of life of patients and their families [3,4]. Questionnaires have been developed and validated in English to assess the quality of life of PCD patients for adults, adolescents aged 13–17 years, children aged 6–12 years, and their caregivers [5,6,7]. The questionnaires were developed based on a multi-stage study involving a literature review, expert discussion, an overview of the available questionnaires, and patient surveys, in collaboration with patients and their caregivers, under Food and Drug Administration (FDA) guidelines, and validated in subsequent years [7,8]. In the following years, the questionnaires were translated into several languages and introduced in many countries, including Cyprus [9], Israel [10], and Brazil [11]. This study aimed to prepare a Polish version of the questionnaires and validate them in specific age groups with the participation of Polish patients with PCD.

## 2. Materials and Methods

### 2.1. Translation into Polish

**Figure 1:**
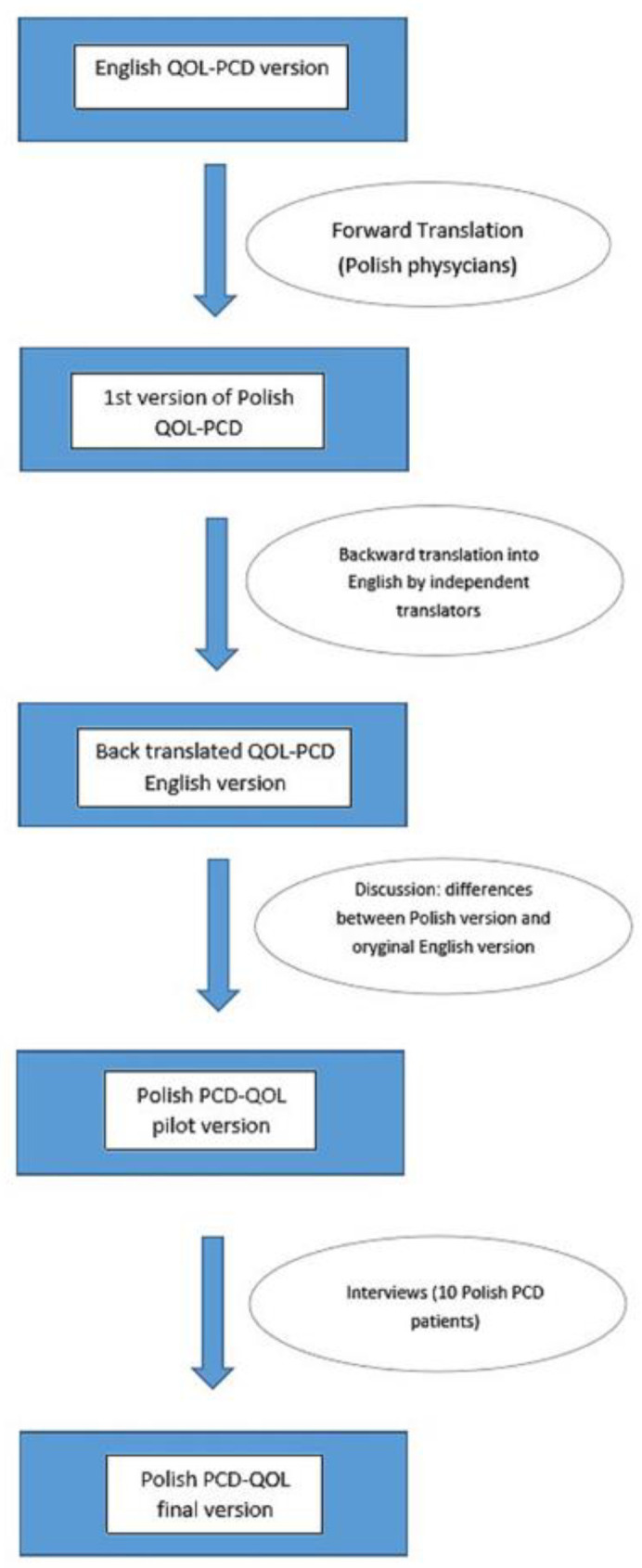
Translation procedure. QOL-PCD—a questionnaire to asses the quality of life of patients with Primary Ciliary Diskinesia.

The individual questionnaires, corresponding to the age groups, were translated from English into Polish by physicians involved in the daily care of PCD patients for whom Polish is the native language and who were fluent in English. Independent translators then translated the material back into English, after which the original versions of the questionnaires were compared with the Polish translations. Any differences were discussed among all the translators, with the participation of the creator of the initial English questionnaire (Dr. Laura Behan). A dozen PCD patients were asked to complete the questionnaires on paper, and the research team discussed the answers to ensure the final versions of the developed questionnaires corresponded to the cultural contexts and that the translations were consistent and understandable to the patients (see Figure 1).

The validation stage of the study was conducted in the form of questionnaires available online between 26 May 2021 and 8 January 2022. The patients completed the questionnaires depending on their age (adults, adolescents, 13–17 years, children, 6–12 years, and caregivers of children aged 6–12 years; the questionnaires are available at the end of the article). Some patients in each group responded twice, two weeks apart (Table 1).

### 2.2. Questionnaires

The questionnaires include between 27 (parent proxy) and 40 questions (adult) and are composed of 7–10 domains (see Table 2). The questionnaires use closed-ended questions with categorical answers and quantitative answers on an ordinal scale: a higher score indicates a better quality of life for the patient.

The average time to complete the questionnaire for all groups was between 5 and 10 min (Table 3), which aligns with the results obtained in the original work for adults [8].

### 2.3. Patient Group Surveyed

The patients were recruited through personal contact by the physicians caring for them daily and with the help of the Polish Ciliary Dyskinesia Society; the prerequisite for joining the survey was a diagnosis of PCD and fluency in Polish. In Poland, the diagnosis of PCD is based on patient’s history, clinical symptoms, measurement of nitric oxide concentration in nasal exhaled air (nasal NO—nNO), the result of an examination of ciliary motility by contrast-phase microscopy (the examination was performed at the Institute of Human Genetics of the Polish Academy of Sciences in Poznań and the Institute of Tuberculosis and Lung Diseases in Rabka-Zdrój), and the assessment of ciliary ultrastructure by electron microscopy and genetic tests.

### 2.4. Statistical Analysis

The validation of the Polish version of the questionnaires was based on the following measurements: internal consistency assessed by Cronbach’s alpha coefficient and split-half reliability, and test–retest reliability of the obtained measurements assessed by the ICC (intraclass correlation coefficient). The statistical analysis was performed using PQStat V.1.8.2 software for Windows (PQStat Software, Poznań, Poland); *p* < 0.05 was considered statistically significant.

Cronbach’s alpha coefficient provides a measure of the internal consistency of a questionnaire. Internal consistency describes the extent to which all the items in a test measure the same concept. The closer the value of Cronbach’s alpha coefficient is to 1, the better the accuracy of the measurement with the evaluated questions. However, a very high Cronbach’s alpha (>0.95) is also undesirable, as it may indicate multidimensionality. The reliability analysis suggests to what extent the particular items measure the qualities that are meant to be evaluated by the questionnaire as a whole. The questionnaire reliability coefficient should be greater than 0.6 and less than 1 [12]. The consistency of the measurements was analyzed using the intraclass correlation coefficient ICC. The ICC value ranges between 0 and 1, where 0 means no consistency, and 1 indicates high similarity between questions from the same group. According to Koo and Li [13], values 0.5 are considered reliable, values > 0.6 are considered good, and values above 0.75 are considered very good.

## 3. Results

Most QOL-PCD domains demonstrated good internal consistency (Table 2). In the adult questionnaire, Cronbach’s alpha was 0.471–0.932 across domains; the lowest internal consistency was observed for vitality, while the highest was for physical functioning. In the parent proxy and adolescent versions of the questionnaires, the lowest internal consistency was observed for lower respiratory symptoms, while the highest was for physical functioning. In the children’s questionnaire, the values of Cronbach’s alpha coefficient were very accurate, i.e., above 0.7 in all domains.

Overall, the internal consistency of all questionnaires was moderate to very good, with Cronbach’s alpha of 0.67–0.91 and split-half reliability of 0.53–0.95 (Table 4). Removing any questions in each questionnaire did not change the value of Cronbach’s alpha.

In the group of patients who completed the questionnaire again after two weeks, we obtained good and excellent repeatability (ICC 0.67–0.91—Table 5).

The overall scale scores of the questionnaire (Table 2) were highest for the adolescent group aged 13–17, with a very high score for the ability to engage in school and daily activities (social functioning: 63.5 and role: 71.5), despite the difficulties associated with the disease. In this area, the lowest scores were shown for the group of adult patients, especially concerning their ability to work and everyday functioning (social functioning: 51.4, role: 45.1). The severity of the disease symptoms was the most troublesome for the adult patients, especially for ear and hearing symptoms (32.0), while the adolescents did not find those symptoms bothersome at all (94.3). All groups of patients and parents perceived upper respiratory symptoms as worse than lower respiratory symptoms. The treatment burden was less severe for the adolescents (59.0) than for the adults (36.7). The assessment of the children aged 6–12 and their caregivers was similar in all domains.

## 4. Discussion

A limitation of the health system is the relatively short time allotted for patients’ follow-up visits in most cases, which means that we rarely talk to patients about how they feel about their disease. Therefore, the use of the QOL-PCD questionnaires might be helpful.

In the adult questionnaire, the median and interquartile range (IQR) results were lower in nearly every domain as compared to the children’s and adolescents’ results. The adult PCD patients perceived the treatment as more burdensome than the other groups, and their social functioning appeared the worst. In each of the comparison groups, the upper respiratory tract symptoms were more troublesome for the patients and the parents than the lower respiratory tract symptoms. Previous studies in adult patients with PCD also indicated that age might influence HRQOL [14].

We found significant differences in most domains of the QOL questionnaires between Polish- and English-speaking patients (Table 6), the former having rated their QOL lower. However, Polish adult patients reported better social functioning, and Polish teenagers and parents reported less troublesome lower respiratory and hearing symptoms.

There are no available publications about the quality of life of Polish PCD patients, but there are some data on Polish patients with cystic fibrosis (CF). CF is a chronic disease affecting various organs, including the respiratory tract. The respiratory symptoms are very similar in CF and PCD patients, and some comparisons of the QOL of patients with these conditions have been published [9]. The parents of PCD patients perceive the treatment as less burdensome than CF patients’ parents (Table 7).

The teenagers in both groups estimate the physical, emotional, and social functioning very similar, and there is a difference in the domains of treatment burden and respiratory symptoms, as PCD patients evaluate those domains worse than their peers with CF. Compared with adult CF patients [15], the quality of life of adult PCD patients is lower in nearly every domain (physical, emotional, and social functioning, health perspective, and respiratory symptoms). The better outcome for CF patients could be explained by a different respondent group (teenagers older than 14 years were included in the group of adults) and the existence of established standards of care and registered CF-specific therapies. It could also be due to a phenomenon called the disability paradox: people with severe and persistent disabilities report that they experience a good or excellent quality of life as an adaptation to the illness or its denial [16].

For the assessment of the original versions of the pediatric, parent proxy, and adolescent PCD-QOL questionnaire, 71 children, 85 adolescents, and 68 parents from multiple centers in the U.K. and North America were recruited and completed age-appropriate questionnaires. In total, 13 children with the same number of parents and 17 adolescents repeated the questionnaire two weeks later to assess its test–retest reliability. It was proven that the pediatric, parent proxy, and adolescent PCD-QOL scales had good internal consistency, good stability across all scales, and validity.

Regarding our research, the children’s PCD-QOL questionnaire had a good outcome for both internal consistency and test–retest reliability. The adolescent questionnaire had a good internal consistency, and the test–retest reliability measured by ICC was under 0.5 for the emotional functioning domain. Compared with the English validation results of the questionnaires [8], worse psychometric results of the parent proxy QOL-PCD questionnaire were observed. This was especially evident in the following domains: treatment burden, social functioning, health perception, eating and weight, and lower respiratory symptoms (*p* > 0.05). The other domains of this questionnaire demonstrated good and ideal internal consistency and good test–retest reliability. The observed poor correlation in particular domains could result from the small number of participants (N = 9).

When comparing our results with those of the English version of the adult QOL-PCD questionnaire, we observed similar psychometric properties, especially regarding the physical functioning subscale, which demonstrated very high internal consistency and test–retest reliability in both validation studies.

Given the almost completely homogeneous ethnic profile of our cohort, the probability of confounding due to cultural differences was limited.

As a study limitation, we have to acknowledge the small study group. PCD is a rare condition, and we collected data from different centers with the ultimate goal of improving patients’ outcomes. A further investigation of the Polish version of the adolescent and parent proxy PCD-QOL questionnaire is required to confirm its good validity and diagnostic accuracy.

## 5. Conclusions

The adult, adolescent (13–17 years), children (6–12 years), and caregiver of children aged 6–12 years versions of the PCD-QOL questionnaire were translated according to international guidelines by independent translators. Differences were discussed among all the translators, with the participation of the creator of the initial English questionnaire. A validated Polish version exhibited moderate to good metric properties in terms of internal consistency and split-half reliability. Due to the small group of patients, parent proxy PCD-QOL needs further investigation.

An essential goal in chronic disease management is to enable the patient to function best in everyday life. The Polish version of the quality of life questionnaire is a simple and feasible tool to assess PCD patients of all ages and can be used during routine follow-up visits. The quality of life of PCD patients is assessed differently depending on the patient’s country of origin. This is the first such report on Polish PCD patients.

## Figures and Tables

**Table 1:** Number of completed questionnaires in each patient group.

Questionnaire	Number of Patients	Number of Patients Who Completed the Questionnaire Twice
Adults aged 18 and over	18	6
Adolescents aged 13–17	8	4
Children aged 6–12	7	4
Parents/Guardians of children aged 6–12	6	3

**Table 2:** Internal consistency of QOL-PCD scales measured by Cronbach’s α and test–retest reliability measured by ICC (intraclass correlation coefficient).

Adults (N = 24)	Items	Scale Median (IQR *)	ICC (95% CI)	Cronbach’s Alpha
Physical functioning	4	55.0 (31.7–81.7)	0.930 (0.872–0.966)	0.932
Vitality	3	54.6 (44.4–61.1)	0.425 (−0.132–0.733)	0.471
Emotional functioning	3	58.3 (45.0–60.0)	0.503 (0.100–0.759)	0.606
Treatment burden	4	36.7 (27.1–47.6)	0.799 (0.621–0.906)	0.806
Social functioning	5	51.4 (41.4–55.5)	0.821 (0.648–0.917)	0.824
Role	4	45.1 (33.3–58.3)	0.594 (0.062–0.824)	0.573
Health perspective	4	53.5 (47.9–60.4)	0.575 (0.169–0.802)	0.618
Upper respiratory symptoms	4	50.7 (33.3–75.0)	0.701 (0.411–0.861)	0.703
Lower respiratory symptoms	6	66.7 (53.3–86.7)	0.839 (0.709–0.922)	0.843
Ears and hearing symptoms	2	32.0 (0–50.0)	0.909 (0.599–0.979)	0.912
**Parents (N = 9)**	**Items**	Scale Median (IQR *)	**ICC (95% CI)**	**Cronbach’s Alpha**
Physical functioning	5	65.9 (40.0–86.6)	0.926 (0.809–0.980)	0.920
Emotional functioning	3	51.8 (44.4–55.6)	0.809 (0.426–0.953)	0.810
Treatment burden	5	51.8 (50.0–58.3)	0.442 (−0.88–0.866) (*p* > 0.05)	0.493
Social functioning	3	68.5 (63.8–72.2)	0.534 (−0.367–0.883) (*p* > 0.05)	0.614
Health Perception	2	50.9 (41.7–58.3)	0 (−1.77–0.46) (*p* > 0.05)	0.404
Eating and weight	3	84.3 (81.9–87.5)	0.438 (−0.836–0.864) (*p* > 0.05)	0.522
Upper respiratory symptoms	4	47.2 (33.3–66.7)	0.656 (0.044–0.912)	0.699
Lower respiratory symptoms	8	65.7 (50.7–79.8)	0.280 (−0.269–0.764) (*p* > 0.05)	0.317
Ears and hearing symptoms	3	79.0 (77.7–100.0)	0.805 (0.392–0.952)	0.848
**Children (N = 11)**	**Items**	Scale Median (IQR *)	**ICC (95% CI)**	**Cronbach’s Alpha**
Physical functioning	5	61.8 (53.3–66.7)	0.760 (0.454–0.924)	0.807
Emotional functioning	4	54.5 (45.8–66.7)	0.631 (0.034–0.891)	0.713
Treatment burden	5	50.3 (30.0–73.3)	0.864 (0.679–0.957)	0.886
Social functioning	4	61.8 (43.3–86.7)	0.842 (0.623–0.951)	0.830
Upper respiratory symptoms	5	45.5(30.0–53.3)	0.577(0.150–0.853)	0.768
Lower respiratory symptoms	6	54.0 (38.9–66.7)	0.668 (0.306–0.890)	0.756
Ear and Hearing Symptoms	3	70.4 (54.2–83.3)	0.626 (0.170–0.879)	0.733
**Adolescents (N = 12)**	**Items**	Scale Median (IQR *)	**ICC (95% CI)**	**Cronbach’s Alpha**
Physical functioning	5	71.3 (66.7–75.0)	0.908 (0.788–0.970)	0.911
Vitality	3	63.2 (60.4–64.6)	0.847 (0.593–0.952)	0.867
Emotional functioning	4	71.9 (70.8–72.9)	0.449 (−0.328–0.822)	0.556
Treatment burden	3	59.0 (55.2–65.6)	0.639 (0.04–0.887)	0.657
Social functioning	4	63.5 (62.0–67.1)	0.630 (0.108–0.881)	0.618
Role	2	71.5 (63.5–78.1)	0.712 (0.236–0.909)	0.694
Upper respiratory symptoms	5	54.2 (42.7–64.6)	0.835 (0.602–0.947)	0.843
Lower respiratory symptoms	6	63.5 (50.0–76.0)	0.540 (−0.27–0.848)	0.506
Ears and Hearing Symptoms	4	94.3 (90.1–98.4)	0.710 (0.300–0.906)	0.899

* Interquartile range.

**Table 3:** Time to complete the questionnaire.

Questionnaire	Average Time to Complete the Questionnaire		
	<5 min	5–10 min	>10 min
Adults aged 18 and over	27%	46%	27%
Adolescents aged 13–17	25%	67%	8%
Children aged 6–12	36%	55%	9%
Parents/Guardians of children aged 6–12	11%	56%	33%

**Table 4:** Analysis of the value of Cronbach’s alpha.

Questionnaire	Cronbach’s Alpha
Adults	0.83
Adolescents aged 13–17	0.81
Children aged 6–12	0.91
Parent proxy (children aged 6–12)	0.67

**Table 5:** Measurement consistency analysis.

Questionnaire	ICC (*p* < 0.05)
Adults	0.83
Adolescents aged 13–17	0.81
Children aged 6–12	0.91
Parent proxy (children aged 6–12)	0.66

**Table 6:** Comparison of QOL among Polish and English PCD children, adolescents, adults, and parents [8,9].

N	Mean Value Domain	English Speaking, PCD-QOL				Polish, PCD-QOL			
		Children	Adolescents	Parent-Proxy	Adults	Children	Adolescents	Parent-Proxy	Adults
1	Physical functioning	79.15	80.95	79.21	80.0	61.8	71.3	65.9	55.0
2	Emotional functioning	72.65	73.82	68.95	86.7	54.5	71.87	51.8	58.3
3	Social functioning	79.71	62.35	79.24	33.3	61.8	63.5	68.5	51.4
4	Treatment burden	56.34	70.82	59.68	66.7	50.3	59.0	51.8	36.7
5	Role	-	80.78	-	66.7	-	71.5	-	45.1
6	Vitality	-	59.22	-	66.7	-	63.2	-	54.6
7	Health Perception	-	-	66.79	50.0	-		50.9	53.5
8	Upper respiratory symptoms	66.48	65.33	55.39	58.3	45.5	54.2	47.2	50.7
9	Lower respiratory symptoms	63.07	58.23	58.66	61.1	54.0	63.5	65.7	66.7
10	Ear and hearing symptoms	74.65	82.64	75.49	66.7	70.4	94.3	79.0	32.0

**Table 7:** Comparison of QOL among Polish CF and PCD children, teenagers, adults, and parents.

Mean Value Domain		CF-QOL				PCD-QOL			
		Children	Teens (12–13 Years)	Parents	>14 Years	Children	Teens (12–17 Years)	Parent-Proxy	Adults
1	Physical functioning	65.03	71.37	76.61	74.73	61.8	71.3	65.9	55.0
2	Emotional functioning	72.30	72.91	67.52	76.77	54.5	71.87	51.8	58.3
3	Social functioning	56.16	69.05	-	69.18	61.8	63.5	68.5	51.4
4	Treatment burden	65.36	70.08	46.35	65.23	50.3	59.0	51.8	36.7
5	Health perspective	-	-	56.84	59.14	-	-	50.8	53.5
6	Respiratory symptoms	71.57	72.12	75.87	66.13	49.75 *	58.85 *	56.45 *	58.7 *

* mean value for upper and lower respiratory tract.

## Data Availability

The data presented in the study are openly available: https://github.com/kotmruczy/PCD-QoL.git (accessed on 18 June 2024). The ownership of the original PCD-QOL Questionnaires in English belongs, among others, to Prof. Jane Lucas (UK) and is available from her (jlucas1@soton.ac.uk).
